# Letter to the Editor Re: Ogawa, Y. *Cancers* 2016, *8*, 28

**DOI:** 10.3390/cancers8060053

**Published:** 2016-05-24

**Authors:** Cameron J. Koch

**Affiliations:** Department of Radiation Oncology, University of Pennsylvania, Philadelphia, PA 19104, USA; kochc@mail.med.upenn.edu; Tel.: +1-215-898-0073

**Keywords:** IR direct effect, IR indirect effect, oxygen effect, radiation sensitizers, radiation protectors

We read with interest the recently published paper by Dr. Ogawa “Paradigm Shift in Radiation Biology/Radiation Oncology—Exploitation of the H_2_O_2_ Effect” for Radiotherapy Using Low-LET (Linear Energy Transfer) Radiation such as X-rays and High-Energy Electrons”. This paper suggests the use of hydrogen peroxide to convert the phenotype of solid tumors from radiation-resistant to radiation-sensitive. This novel approach could have wide application if the distribution of peroxide could be made sufficiently uniform to re-oxygenate cells that were resistant due to hypoxia and Dr. Ogawa additionally suggests that inactivation of peroxidases (e.g., glutathione peroxidase and catalase) by the hydrogen peroxide will itself cause sensitization [1]. 

This is certainly an area of radiation research that needs more study, but this letter's purpose is to question some of the radiation chemistry principles described that are at odds with a great deal of experimental research dating from the 1970s and earlier. There are three areas of concern: (1) confusion and mixing of the concepts of endogenous radiation sensitivity due to DNA damage with that for specialized cells such as lymphocytes. The latter are radiosensitive and die primarily by apoptosis, possibly mediated, at least in part, by membrane damage whereas the former die primarily due to chromosome damage; (2) the physical-chemical mechanism underlying the direct *versus* indirect effect; and (3) the mechanism of radiosensitization by oxygen.

The mechanisms of apoptotic death for irradiated lymphocytes are not entirely clear, since apoptosis can arise from either DNA damage or membrane damage, and the latter has been implicated over certain dose ranges even for more resistant tumor cells [2]. Membrane damage may be partially mediated by radiation-induced peroxides, but the initiating events are still thought to be hydroxyl radicals, not radiation-induced peroxides [3]. A common misstatement that one finds scattered throughout the modern literature (far too often for citation), is that reactive oxygen species or ‘ROS’, including peroxides, can be lumped together with radiation as if their mechanisms of action were similar. In fact, an enormous body of work has shown that clonogenic cell-death from radiation is mediated by (clusters of) hydroxyl radicals [4,5,6,7]. Although a hydroxyl radical can be considered one of the reactive species derived from the reduction of oxygen (3-electron reduction product, after superoxide radical and hydrogen peroxide), the hydroxyl radicals critical to the efficacy of radiation are derived from water radiolysis, not oxygen reduction. The amount of hydrogen peroxide produced by 10 Gy is only about 3 µM under aerobic conditions, similar to the hydroxyl radical yield [8]. However, at tissue densities, one requires 10s or 100s of millimolar hydrogen peroxide for toxicity, and this is due to the extensive peroxide detoxification of tissues that endogenously contain multi-millimolar glutathione as well as the dismutating enzyme catalase, which directly converts hydrogen peroxide to water and oxygen. Upon oxidation by GSH-peroxidase, the resulting oxidized glutathione (GSSG) can be continuously reduced with recycling of GSH through the action of the pentose cycle [9,10]. For cells in tissue culture, hydrogen peroxide can appear to be toxic at low concentrations when the cell density is very low, but not before each cell processes of the order of 100–200 mM of it [11,12].

As indicated above, radiation-induced tumor-cell toxicity is primarily mediated through DNA damage [13]. In his review, Ogawa discusses DNA damage *vis-a-vis* the indirect effect and the oxygen effect as being directly connected—this view is not supported by the literature. With respect to the first of these, the indirect effect is defined as originating from water radiolysis. A figure from a current textbook [14] (Figure 1, as modified in the Ogawa paper [1]) illustrates the DNA damaging events in cartoon style (little “poofs”), without any specific chemical basis, but properly associates the indirect effect as originating from OH• due to water radioloysis, while the source of the electron for the direct-effect ionization arises through scattering by another molecule. Unfortunately, the next version of this figure (Ogawa, Figure 2 [1]) depicts the direct effect as arising from an interaction of an incoming photon with the DNA (an unlikely source) and eliminates the water-radiolysis-derived hydoxyl intermediate from the indirect effect. It further indicates hydroxyl radicals originating in the mitochondria and cytoplasm as being of importance. Since hydroxyl radicals react at diffusion-controlled rates, their production in the cytoplasm or elsewhere is of no consequence (established many decades ago using site-specific isotope decay [15]). 

When the indirect effect produces hydroxyl radicals immediately adjacent to DNA, they can extract a hydrogen atom from sugars and bases leaving a carbon centered neutral radical on the DNA target. It is not immediately obvious that the direct effect produces a similar species, because if an electron is ejected from DNA through interaction with another electron, the resulting positive radical is typically a very strong acid [8]. Thus, the essentially immediate release of a hydrogen ion at neutral pH then leads also to a neutral carbon-centered radical. So while there are many subtleties to this simple picture, including the formation of methyl-peroxy radicals by the DMSO scavenger, OH• addition, molecular excitation rather than ionization, *etc*., it is simply not true that there is no indirect effect under hypoxic conditions [16,17], or that oxygen cannot interact with radicals produced by either mechanism. 

The oxygen effect can be well modeled by a simple chemical mechanism: target radical oxidation by oxygen (or sensitizers) resulting in further oxidation and more damage, in competition with target radical reduction by thiols, leading to less damage. This extremely simple picture (competition model) was first suggested by the French scientist Prevot-Bernas [18], and was subsequently found to hold true over many decades of modifier concentration by the present writer [19]. Wardman’s excellent summary of the ‘oxygen effect’ discusses the fact that thiols cannot actually rescue the initial structure [20]—nevertheless, from the point of view of final impact on the cells’ colony-forming ability, the competition model represents an extremely good approximation. In the same review, it was shown that chemical sensitizers were not nearly as efficient as oxygen, and this formed part of an interesting discussion [21]. In that discussion, the extremely poor performance of hydrogen peroxide as a sensitizer was noted. Thus, we are unaware of any data that suggest hydrogen peroxide, or inhibiting the action of peroxidases, has anything to do with the oxygen effect or the indirect effect, and no references on the same subject were given for the alternate suggestion by Ogawa [1]. It had often been suggested that the lack of an oxygen effect for DNA damage in solution cast doubt on the competition model. However, we showed that if one roughly enabled the equivalent cellular scavenger levels (using glycerol) and provided a suitable thiol competitor (GSH) the sensitivity of DNA in solution very closely resembled that in the cell [22]. This work also showed that the glycerol scavenger of the indirect effect worked almost as well under hypoxic conditions as aerobic conditions.

In the final modification of the figure from Hall and Giaccia (Ogawa; Figures 2 and 3 [1]) hydrogen peroxide is seen to originate from hydroxyl radicals, which then react to produce more hydroxyl radicals via a “Fenton” reaction. In ultrapure water and at high dose rates, it is certainly possible for two hydroxyl radicals to make hydrogen peroxide [8], but this should by no means be considered a reaction appropriate to the cell, where essentially all hydroxy radicals will react with organic molecules and where hydrogen peroxide arises from electron capture by oxygen, with subsequent dismutation of the superoxide radical by superoxide dismutase. The Fenton reaction is an example of a reaction that has been analyzed in great detail by radiation chemists [23] but is often misunderstood by “amateurs”. It can hardly be considered as a mainstream player in the radiation response of cells. This confusion is exemplified by Wikipedia’s description of the Fenton reaction, showing hydrogen peroxide as both an oxidant of Fe^2+^ and a reductant of Fe^3+^, producing radicals in both directions without considering the pH or kinetic constants. 

To summarize, it seems unfortunate that Radiation Chemistry is such a mature and poorly funded field—but it is essential that we retain the lessons of the past and not start over in attempts to understand very basic aspects of radiation biology. This is not to understate the potential value of the use of peroxide in therapy, as suggested by Dr. Ogawa. Rather, the need for some good free-radical and peroxide-based chemistry is critically needed to move forward with this modality. This is unlikely to be an easy task due to the extreme complexities of interactions between exogenous and endogenous reactive oxygen and nitrogen species [24]. As a start, the fact that oxygen-bubble emboli are being produced by the Kortuc protocol (using 3% peroxide, which is about 1 molar) is good evidence that catalase and other peroxidases are active in this setting [1].

## Abbreviations

The following abbreviations are used in this manuscript:

GSH: reduced glutathione
GSSG: oxidized glutathione
